# Who is mentally healthy? Mental health profiles of Japanese social networking service users with a focus on LINE, Facebook, Twitter, and Instagram

**DOI:** 10.1371/journal.pone.0246090

**Published:** 2021-03-03

**Authors:** Ryota Sakurai, Yuta Nemoto, Hiroko Mastunaga, Yoshinori Fujiwara

**Affiliations:** 1 Research Team for Social Participation and Community Health, Tokyo Metropolitan Institute of Gerontology, Tokyo, Japan; 2 Japan Support Center for Suicide Countermeasures, National Center of Neurology and Psychiatry, Kodaira, Tokyo, Japan; Indiana University Bloomington, UNITED STATES

## Abstract

**Background:**

Both negative and positive associations between social networking service (SNS) usage and mental health have been suggested by previous studies; however, their differences by type of SNS and age remain unclear. We addressed this issue based on the frequency of traditional communication such as face-to-face and non-face-to-face communication (e.g., phone, email, and letters).

**Methods:**

In total, 8,576 individuals participated, including 2,543 aged 18–39, 3,048 aged 40–64, and 2,985 aged over 65 years. They were asked to indicate their frequency of SNS usage, both for posting and checking, of LINE (a popular message application in Japan), Facebook, Twitter, and Instagram, with frequent usage defined as posting or checking more than a few times a week. To determine mental health status, WHO-5 (i.e., well-being), K6 (i.e., distress symptoms), and feelings of loneliness were assessed. Multiple and logistic regression analyses were adjusted for the frequency of traditional communication. To avoid type 1 error, a Bonferroni correction of *p* ≤ 0.002 was applied in the regression models (*p* = 0.05/18, a number of regression models).

**Results:**

The most frequently used SNS across the three age groups was LINE; frequent usage (both posting and checking) among older adults was independently associated with better well-being. Frequent posting on Facebook was associated with better well-being in middle-aged adults. Young adults who frequently checked on Instagram showed a tendency toward better well-being and lower distress symptoms. On the contrary, frequent usage of Twitter was associated with distress symptoms or feelings of loneliness across all three age groups.

**Conclusions:**

We found generational and SNS-type-dependent negative and positive associations between SNS use and mental health, indicating the possible influences of SNS use and the importance of non-SNS communication.

## Introduction

Social interactions and ties are fundamental components of human life and play an important role in maintaining health. A previous meta-analysis in fact showed that individuals who are more firmly embedded in their social surroundings are healthier than those with relatively thin social ties, an effect that may exceed the effects of other lifestyle factors such as smoking, drinking, and exercise habits [1]. This health impact is likely to be prominent in older adults who are predisposed to functional impairments with aging. Indeed, social isolation, which is an objective and quantifiable reflection of a reduced social network size and lack of social contact, among older adults increased the risk for subsequent all-cause mortality regardless of race [2–4], even among individuals without any physical dysfunction at the baseline [4]. These findings suggest that facilitating social interactions that reduce social isolation can be considered an important health measure, which can be applied globally.

A major current focus on preventing health issues due to poor social networks is whether online social interaction helps maintain mental health. A social networking service (SNS), denoting web-based services used to create social interactions, seems to be a useful tool to communicate and share one’s emotions and thoughts with others. Some studies have found beneficial effects of SNS on social capital, social support, and relationship maintenance [5–7], which probably result in sound mental health. However, other studies cast doubt on this prospect; it has been indicated that SNS induced stressful experiences, with the users showing poor well-being [8, 9]. A recent meta-analysis also reported that the adolescents were found to show an association between greater time spent on SNS and higher levels of depression [10].

These conflicting results regarding SNS usage and mental health may be due to the failure to examine the respective frequencies of traditional face-to-face and non-face-to-face communications (e.g., telephone, e-mail, letters). If individuals who have decent social interactions with others use traditional communication channels, they may presumably not be influenced by the negative effect of SNS. Considering that the frequency of traditional communication such as face-to-face and non-face-to-face communication is strongly related to mental health, it is expected that traditional communication confounds (diminishes) the association between SNS usage and mental health.

Furthermore, the types of SNS and their usage (i.e., posting or checking) may also confound the positive and negative effects of SNS. A recent survey investigating the association between the usage of five types of SNS (i.e., YouTube, Twitter, Facebook, Snapchat, and Instagram) and mental health among young adults showed that Instagram was ranked the worst SNS for their young people’s mental health [11]. However, extensive findings regarding the effects of SNS on mental health are limited to the findings mainly focusing on either total SNS usage or each SNS usage alone, particularly focusing on young adults [10, 12–14]; thus, the differences in the influence of the type of SNS remains an open research question.

Another pertinent question is the generational differences in SNS usage and its effects. Of special interest are the prevalence and characteristics of SNS usage among older adults. Some studies of older adults and SNS have focused on the usability analysis and case studies on online social networks [15–19]. Although a previous study suggested an association between higher frequency of technology usage for social connection (defined by the total usage of e-mail, SNS [e.g., Facebook or Twitter], and online video or phone calls [e.g., Skype]) and better mental health [20], the influence of the type of SNS on mental health among older individuals and the confounding effect of traditional communication on such association have been poorly known. Indeed, the literature concerning the association of SNS usage with mental health has been mainly limited to adolescents [10, 12].

As outlined, there has been no consensus on the generational and SNS-type-dependent negative and positive associations between SNS use and mental health. In this study, we sought to extend the current findings by examining the mental characteristics of people who regularly use SNS. More specifically, we focused on the posting and checking on LINE (a popular message application in Japan), Facebook, Twitter, and Instagram for three generations (young, middle-aged, and older adults) to elucidate whether (1) the association between SNS usage and mental health varies by the type of service and generation, and (2) whether each association is independent of the frequency of traditional communication, such as face-to-face and non-face-to-face communication (e.g., phone, email, and letters), after taking into account the possession rate of communication devices for SNS purposes. The findings of the present study may contribute towards balancing the potential benefits and harms of social media use.

## Materials and methods

### Participants

Data were collected from the second wave of an ongoing prospective survey intended to assess the health policy of a municipality. From September to November 2019, we conducted a mail survey of residents aged 18 years and older in a town located in central Tokyo, Japan (with a working-age population ranging between 15 and 64 years constituting 168,925 people and an old-age population of 65 years and above constituting 56,378 people as of April 2019) based on the basic resident register. The sample of this survey was randomly selected, excluding people of foreign nationality, institutionalized older adults, and those who had been registered as needing above Care Level 4 of the national long-term care insurance system (i.e., considered dependent in the basic activities of daily living, such as toileting or feeding). We also sent a questionnaire to 3,842 individuals who responded in a baseline survey in 2016 that was conducted in the same manner as the present survey. In total, we sent a questionnaire to 21,300 individuals (3,842 previous respondents in the first wave and 17,458 newly selected sample) in the current survey.

The study was conducted in accordance with the ethical standards outlined in the Declaration of Helsinki. Ethics approval was obtained from the Tokyo Metropolitan Institute of Gerontology. Informed consent was obtained at enrollment in accordance to the protocols approved by the local institutional review board.

### Questionnaires

#### Possession of communication devices and SNS usage

First, the possession rates of communication devices, including a flip phone, smartphone, tablet, and computer were assessed, and having a device except the flip phone was used to define the holder of a communication device for SNS purposes. Participants were then asked about their frequency of usage of each SNS, including LINE, Facebook, Twitter, and Instagram, separately. The question on usage was divided into posting and checking. The responses were categorized as: (1) *every day*, (2) *a few times a week*, (3) *a few times a month*, and (4) *no use*. A frequent usage of each SNS was defined as usage of more than a few times a week (see S1 Table).

#### Mental health

In the present study, we defined mental health status using WHO-5, K6, and feelings of loneliness. WHO-5 is a short generic global rating scale measuring subjective well-being. The scale has adequate validity as both a screening tool for depression and an outcome measure in clinical trials for Japanese samples [21]. The respondents were asked to rate how well each of the five statements applied to them within the last 14 days. Each of the five items is rated from 5 (*all of the time*) to 0 (*none of the time*). The scores for these subscales ranged from 0 to 25, with lower scores indicating lower well-being.

K6, which contains six items on depression and anxiety, is a previously validated questionnaire for measuring distress symptoms among Japanese sample, including overall worries and more somatic complaints [22, 23]. The respondents were asked to rate how well each of the six statements was applied within the last 30 days. Response categories included five categories from *all of the time* to *none of the time*, and the total score ranged from 0 to 24, with higher scores indicating greater distress and depressive symptoms.

Feelings of loneliness were measured using a single item, which is widely used [24]: “How often do you feel isolated from the community?” and the responses were categorized as (1) *very often*, (2) *quite often*, (3) *not very often*, and (4) *never*. Participants were then assigned to either the loneliness (*very often* and *quite often*) or non-loneliness (*not very often* and *never*) groups. It has been suggested that the lack of perception of being socially connected results in loneliness [25], which is a well-known risk factor for adverse health outcomes.

#### Covariates for the association between SNS usage and outcomes

Gender, age, level of education (low-level education ≤ 9), living arrangement (living alone or not), presence of chronic disease, subjective health, subjective financial status, frequency of going outdoors, and frequency of traditional communication with others were introduced as covariates. The level of instrumental activity of daily living (IADL), an indicator of the ability to live independently in a community, was assessed on only older adults as a covariate.

The presence of chronic disease was assessed, and those with at least one chronic disease (e.g., hypertension, heart disease, cerebrovascular disorder, and diabetes mellitus) were defined as having a chronic disease. Subjective health was assessed as *excellent*, *good*, *fair*, or *poor*; participants were then assigned to either the good (*excellent* or *good*) or poor (*fair* or *poor*) groups. Their subjective financial situation was determined based on a method used in a previous study [26]. Participants were asked to respond to a question regarding financial leeway with five choices, ranging from *I have much financial leeway* to *My finances are very tight*. Participants were then classed as having a moderate (*I have much financial leeway* to *Average*) or poor (*My finances are tight* to *My finances are very tight*) financial situation. For the frequency of going outdoors, high (*go out every day*) or low (*go out every few days or less*) frequency of going outdoors daily was assessed. IADL for older adults was evaluated using the IADL subscale of the Tokyo Metropolitan Institute of Gerontology Index of Competence [27], a questionnaire assessing the ability to (1) use public transportation independently, (2) shop for daily necessities, (3) prepare meals independently, (4) pay bills, and (5) manage banking independently.

For the frequency of traditional communication with others, the participants were asked about the frequency of both face-to-face and non-face-to-face (e.g., telephone, e-mail, letters) interactions with others, in addition to those practiced through SNS. The responses were then categorized as (1) *every day*, (2) *4–5 times a week*, (3) *2–3 times a week*, (4) *once a week*, (5) *2–3 times a month*, (6) *once a month*, (7) *less than once a month*, or (8) *no contact*. Based on previous studies, we defined poor traditional communication (i.e., social isolation) as having contact with anyone for less than once a week [4, 28–30].

### Data analysis

We classified the possession rate of a communication device into the following three groups: having a communication device for SNS purposes, having a communication device for non-SNS purposes, and having nothing. Similarly, the frequency of usage of each SNS (LINE, Facebook, Twitter, and Instagram) was summarized to confirm the rate of frequent usage of each SNS.

Multiple regression analyses of poor well-being (assessed by WHO-5) and distress symptoms (assessed by K6) and logistic regression analysis on the feelings of loneliness were run separately for three age groups (i.e., young adults aged 18–39, middle-aged adults aged 40–64, and older adults aged over 65 years) to assess the association with frequent usage of each SNS, separately for checking and posting. Each mental health variable was the dependent variable and each SNS usage was the independent variable; in total, we ran 18 models (three dependent variables by three age groups via two types of usage [posting and checking]). The percentage of participants for whom covariates were missing across each variable ranged from 0 to 3.9% for young adults, 0 to 2.6% for middle-aged adults, and 0 to 4.1% for older adults. The percentages of those with one or more missing measurements were 8.7% for young adults, 7.3% for middle-aged adults, and 14.5% for older adults. Missing values among covariates were imputed by multiple imputation, which allowed us to draw inferences for multivariate estimates from a dataset with missing values, with fully conditional specification. The imputation model consisted of the variables included in the regression analyses described above; 50 imputed datasets with missing values were created and regression analyses were then performed separately for each age group. Each model was adjusted for gender, age, level of education, living arrangement, comorbidity, subjective health, subjective financial status, frequency of going outdoors, IADL level (only in older adults), and poor traditional communication. In this case, participants who did not have any communication devices for SNS purposes were regarded as those who never used a given SNS. To avoid type 1 error, a Bonferroni correction of *p* < 0.002 was applied in the regression models (*p* = 0.05/18, a number of regression models). All statistical analyses were performed using PC-compatible IBM SPSS version 23.0 (SPSS Inc., Chicago, IL, USA).

## Results

### Respondents’ characteristics, possession rate of communication device, and SNS usage

**Fig 1:**
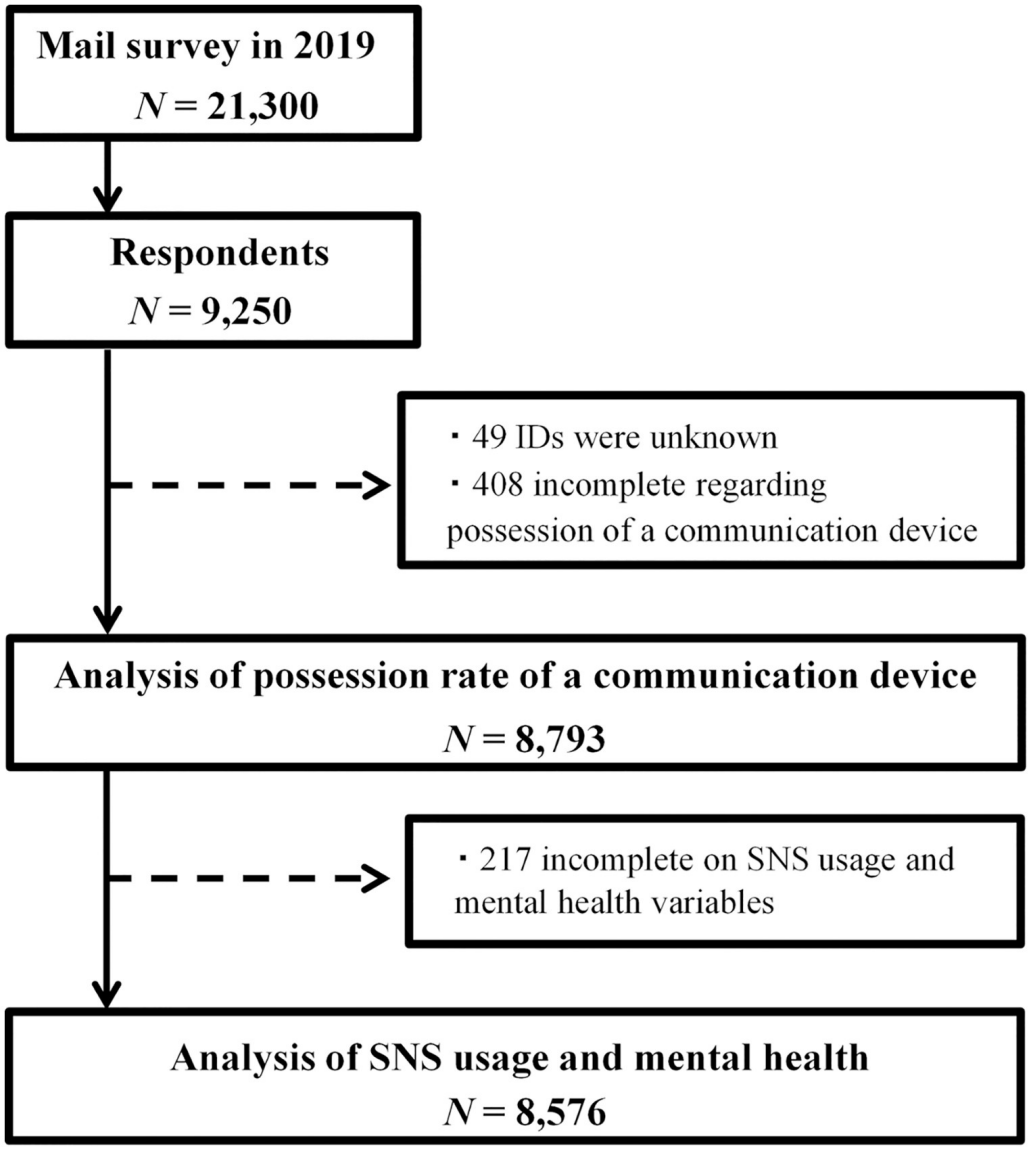
Schematic diagram of the selection of study participants.

Of the 21,300 questionnaires mailed, 9,250 responded in the survey (response rate, 43.4%), of which 49 constituted unparseable data (e.g., the identification number was broken). We excluded 408 participants owing to incomplete data regarding the possession of a communication device. In total, 8,793 respondents were analyzed for the possession rate of a communication device. Furthermore, 217 of these were incomplete on SNS usage and main outcomes (i.e., WHO-5, K6, and feelings of loneliness), and thus 8,576 participants, consisting of 2,543 young, 3,048 middle-aged, and 2,985 older adults, were included in the analysis of the mental health profiles of SNS users (Fig 1).

Table 1 shows the demographic and lifestyle characteristics of respondents by age group. There was not much difference in the gender ratio among the three age groups, and the mean age of each age group was 29.7 for young adults, 51.9 for middle-aged adults, and 74.7 for older adults. Overall, our participants had a high education level. While the percentage of participants with each chronic disease tended to be higher in the senior group, the percentage of participants with poor mental health (i.e., poor well-being, distress symptoms, and feelings of loneliness) tended to be higher in the younger group.

**Table 1:** Characteristics of the analysis objects of SNS usage.

Variables	Young adults (aged 18–39 yr.)		Middle-aged adults (aged 40–64 yr.)		Older adults (aged 65–97 yr.)	
	*n*	Statistics	*n*	Statistics	*n*	Statistics
**Female, *n* (%)**	2543	1524 (59.9)	3048	1697 (55.7)	2985	1632 (54.7)
**Age, mean (*SD*)**	2543	29.7 (6.7)	3048	51.9 (7.1)	2985	74.7 (6.6)
**Low-level education**, ^a^ ***n* (%)**	2445	47 (1.9)	2969	58 (2.0)	2861	428 (15.0)
**Living alone, *n* (%)**	2543	312 (12.3)	3048	322 (10.6)	2985	567 (19.0)
**Having chronic disease, *n* (%)**	2464	327 (13.3)	2976	1052 (35.3)	2917	2132 (73.1)
**Poor communication**, ^b^ ***n* (%)**	2521	660 (26.2)	3011	973 (32.3)	2882	621 (21.5)
**Subjective health, poor, *n* (%)**	2523	252 (10.0)	3026	451 (14.9)	2877	693 (24.1)
**Low frequency of going outdoors**, ^c^ ***n* (%)**	2536	323 (12.7)	3035	400 (13.2)	2952	1133 (38.4)
**Subjective financial status, poor, *n* (%)**	2501	603 (24.1)	3009	746 (24.8)	2917	587 (20.1)
**WHO-5 (/25), mean (SD)**	2543	13.2 (5.4)	3048	12.9 (5.4)	2985	14.2 (5.6)
**K6 (/24), mean (SD)**	2543	5.3 (5.0)	3048	4.5 (4.3)	2985	4.1 (4.0)
**Having feelings of loneliness, *n* (%)**	2543	424 (20.0)	3048	468 (18.1)	2985	398 (15.4)

^a^ Years of education ≤ 9.

^b^ Individuals who had contact less than once a week with others through traditional communication, such as face-to-face and non-face-to-face (e.g., telephone, e-mail, letters) communication.

^c^ Individuals who went out once every 2–3 days or less.

**Fig 2:**
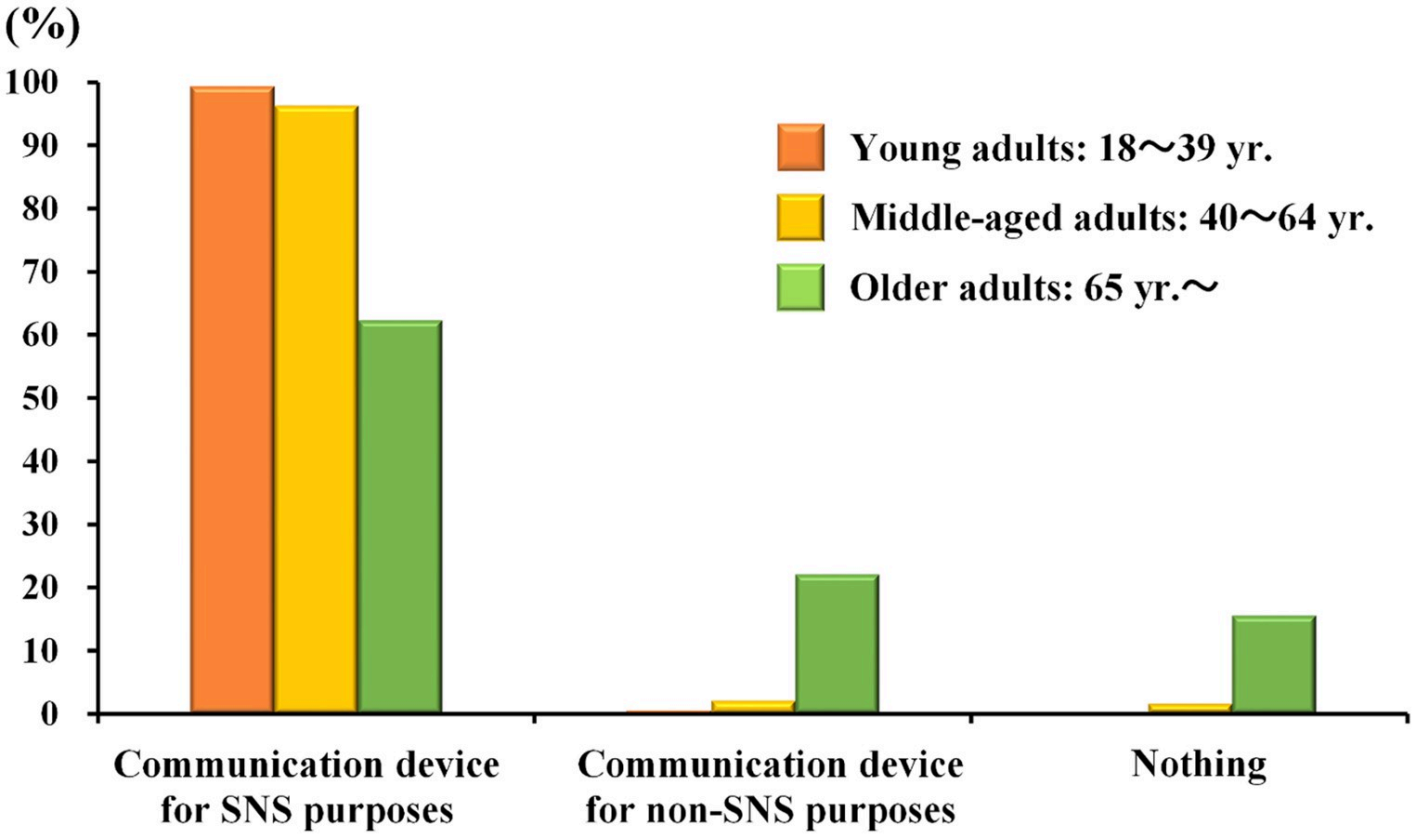
Possession rate of communication devices for SNS purposes across three generations. *Communication device for non-SNS purpose includes individuals who have only flip phones.

Fig 2 shows the possession rate of a communication device that can use SNS by age group. More than 95% of young and middle-aged adults had a communication device for SNS purposes. Furthermore, even older adults showed a relatively high possession rate (62.3%), whereas the possession rate gradually decreased after age 70 (35% for those in their 80s; see S1 Fig).

**Fig 3:**
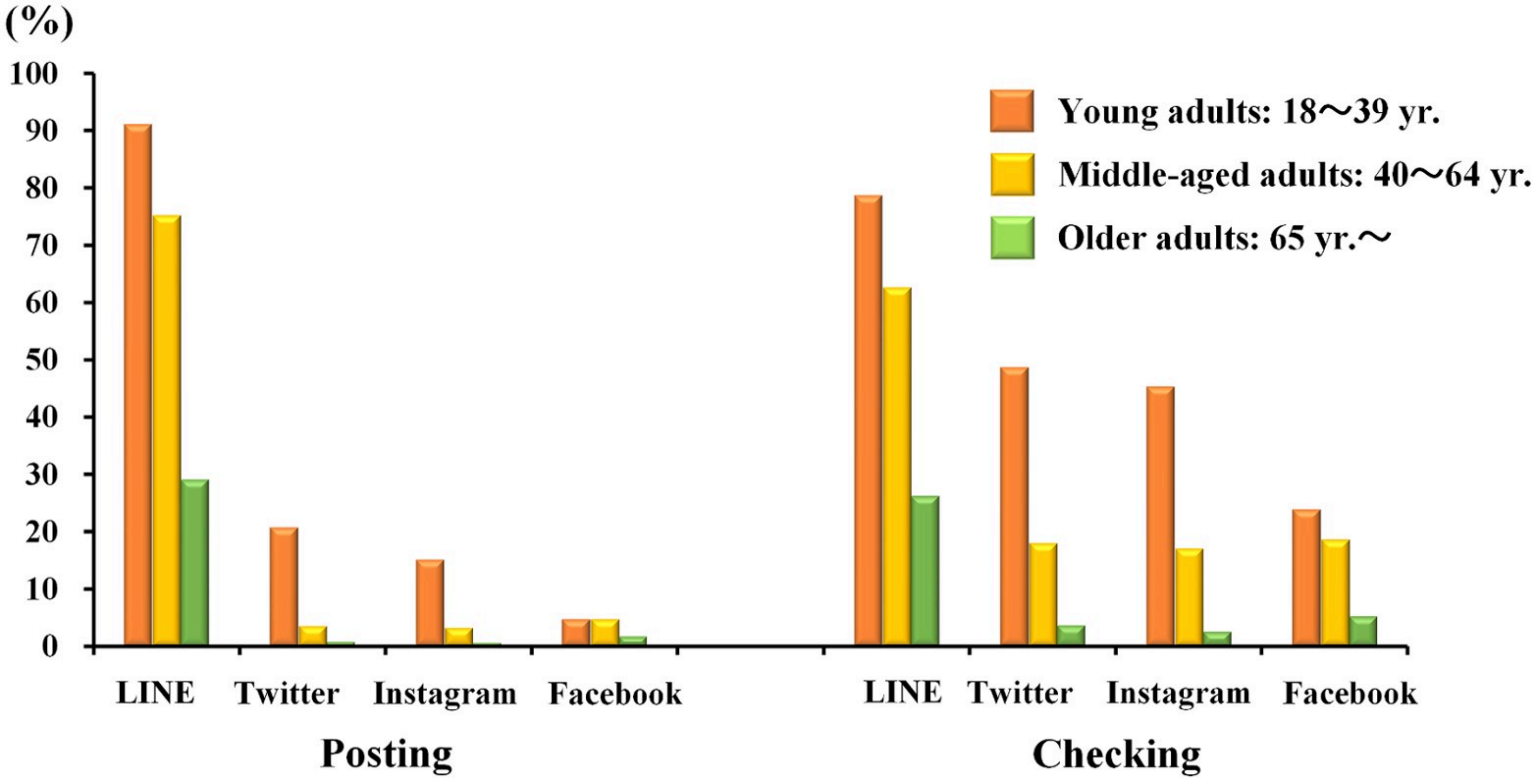
Prevalence of frequent SNS users for each SNS application across three generations. *Frequent usage of each SNS was defined as usage of more than a few times a week. Infrequent users include participants who did not have any communication devices for SNS purposes.

Fig 3 shows the prevalence of SNS usage. The most frequently used SNS was LINE, both in terms of posting and checking among all generations, followed by Twitter, Instagram, and Facebook. This is true for those of older age, and half of those in their 60s and one-third of those in their 70s used LINE (see S2 Fig). Only middle-aged adults showed higher Facebook usage than Twitter usage.

### The association between SNS usage and well-being

Table 2 shows the results of the multiple regression model for the association between SNS usage and well-being status, which is assessed by WHO-5 for three age groups.

**Table 2:** Multiple regression analysis examining the association between SNS usage^†^ and poor well-being detected by WHO-5.

	Young adults (n = 2543)		Middle-aged adults (n = 3048)		Older adults (n = 2985)	
Independent variables	B (95% CI)	*p*-value	B (95% CI)	*p*-value	B (95% CI)	*p*-value
***Posting***						
**LINE**	0.59 (-0.15, 1.33)	.118	0.54 (0.11, 0.97)	.015	0.85 (0.41, 1.29)	**< .001**
**Facebook**	1.45 (0.47, 2.43)	.004	1.00 (0.15, 1.84)	**.002**	2.14 (0.57, 3.70)	.007
**Twitter**	-0.52 (-1.05, 0.02)	.059	0.41 (-0.59, 1.41)	.423	0.35 (-2.14, 2.85)	.782
**Instagram**	0.89 (0.27, 1.51)	.005	0.78 (-0.31, 1.87)	.161	-0.85 (-3.75, 2.06)	.568
**Poor communication**^††^	-1.67 (-2.14, -1.19)	**< .001**	-1.71 (-2.11, -1.32)	**< .001**	-1.40 (-1.87, -0.92)	**< .001**
***Checking***						
**LINE**	0.51 (0.00, 1.02)	.050	0.25 (-0.13, 0.63)	.193	0.97 (0.51, 1.42)	**< .001**
**Facebook**	0.28 (-0.21, 0.78)	.263	0.54 (0.04, 1.05)	.035	1.00 (0.05, 1.95)	.039
**Twitter**	-0.21 (-0.65, 0.22)	.340	0.00 (-0.51, 0.50)	.990	0.26 (-0.85, 1.37)	.643
**Instagram**	0.89 (0.43, 1.36)	**< .001**	0.66 (0.10, 1.21)	.020	0.05 (-1.36, 1.46)	.941
**Poor communication**^††^	-1.63 (-2.10, -1.15)	**< .001**	-1.72 (-2.11, -1.32)	**< .001**	-1.40 (-1.87, -0.92)	**< .001**

B indicates unstandardized coefficient. Significant values after applying the Bonferroni correction are in bold.

^**†**^ A value of 1 was given to frequent usage, 0 to infrequent usage.

^††^ Individuals who had contact less than once a week with others through traditional communication, such as face-to-face and non-face-to-face (e.g., telephone, e-mail, letters) communication. A value of 1 was given for poor communication, 0 for non-poor communication.

Each regression model, which was adjusted for covariates (i.e., gender, age, level of education, living arrangement, presence of chronic disease, subjective health, subjective financial status, frequency of going outdoors, frequency of traditional communication with others, and the level of instrumental activity of daily living [only for the group of older adults]), was performed separately for posting and checking on SNS for each age group. All SNS usages were introduced in the same model as the independent variables.

Frequent checking of Instagram among young adults was independently associated with higher well-being (B: 0.89; 95% confidence interval [CI]: 0.43, 1.36; *p* < .001). Middle-aged adults who frequently posted on Facebook showed higher well-being, which is independent of the frequency of traditional conversation (B: 1.00; 95% CI: 0.15, 1.84; *p* = .002). For older adults, both frequent posting and checking on LINE were independently associated with better well-being (B: 0.85; 95% CI: 0.41, 1.29; *p* < .001 and β: 0.97; 95% CI: 0.51, 1.42; *p* < .001).

### The association between SNS usage and distress symptoms

Table 3 shows the results of the multiple regression model for the association between SNS usage and distress symptoms assessed by K6 for three age groups. Frequent checking on Instagram was independently associated with lower distress symptoms (B: -0.88; 95% CI: -1.30, -0.45; *p* < .001), whereas both frequent posting and checking on Twitter were associated with greater distress symptoms among young adults (B: 0.83; 95% CI: 0.34, 1.32; *p* = .001 and B: 0.72; 95% CI: 0.33, 1.12; *p* < .001). Middle-aged adults who frequently posted on LINE showed a tendency toward lower distress symptoms (B: -0.52; 95% CI: -0.87, -0.16; *p* = .001). Significant associations between frequent posting or checking on Twitter and greater distress symptoms was observed, independent of the frequency of traditional conversation (B: 0.98; 95% CI: 0.15, 1.80; *p* = .002 and B: 0.75; 95% CI: 0.34, 1.17; *p* < .001), similar to the young adults. There were no significant associations between SNS usage and distress symptoms assessed by K6 for older adults.

**Table 3:** Multiple regression analysis examining the association between SNS usage^†^ and distress symptoms detected by K6.

	Young adults (n = 2543)		Middle-aged adults (n = 3048)		Older adults (n = 2985)	
Independent variables	B (95% CI)	*p*-value	B (95% CI)	*p*-value	B (95% CI)	*p*-value
***Posting***						
** LINE**	-0.76 (-1.43, -0.09)	.026	-0.52 (-0.87, -0.16)	**.001**	-0.27 (-0.59, 0.06)	.106
** Facebook**	-0.22 (-1.11, 0.67)	.630	0.07 (-0.63, 0.76)	.854	-0.92 (-2.04, 0.21)	.109
** Twitter**	0.83 (0.34, 1.32)	**.001**	0.98 (0.15, 1.80)	**.002**	1.87 (0.04, 3.69)	.045
** Instagram**	-0.52 (-1.08, 0.04)	.070	0.66 (-0.23, 1.54)	.148	0.82 (-1.32, 2.95)	.455
**Poor communication**^††^	1.05 (0.61, 1.48)	**< .001**	0.72 (0.40, 1.04)	**< .001**	0.63 (0.28, 0.99)	**< .001**
***Checking***						
** LINE**	-0.39 (-0.85, 0.08)	.103	-0.19 (-0.50, 0.13)	.241	-0.31 (-0.65, 0.02)	.066
** Facebook**	-0.08 (-0.53, 0.37)	.723	0.03 (-0.38, 0.44)	.882	-0.29 (-0.98, 0.40)	.411
** Twitter**	0.72(0.33, 1.12)	**< .001**	0.75 (0.34, 1.17)	**< .001**	0.47 (-0.35, 1.28)	.259
** Instagram**	-0.88 (-1.30, -0.45)	**< .001**	-0.27 (-0.72, 0.18)	.235	0.17 (-0.85, 1.18)	.748
**Poor communication**^††^	1.02 (0.59, 1.44)	**< .001**	0.75 (0.43, 1.07)	**< .001**	0.62 (0.27, 0.98)	**.001**

B indicates unstandardized coefficient. Significant values after applying a Bonferroni correction are in bold.

^**†**^ A value of 1 was given to frequent usage, 0 to infrequent usage.

^††^ Individuals who had contact less than once a week with others through traditional communication, such as face-to-face and non-face-to-face (e.g., telephone, e-mail, letters) communication. A value of 1 was given for poor communication, 0 for non-poor communication.

Each regression model, which was adjusted for covariates (i.e., gender, age, level of education, living arrangement, presence of chronic disease, subjective health, subjective financial status, frequency of going outdoors, frequency of traditional communication with others, and the level of instrumental activity of daily living [only for the group of older adults]), was performed separately for posting and checking on SNS for each age group. All SNS usages were introduced in the same model as the independent variables.

### The association between SNS usage and feelings of loneliness

Table 4 shows the results of the logistic regression model for the association between SNS usage and feelings of loneliness by age group. Compared to those with infrequent usage, frequent posting and checking of Twitter among middle-aged adults were both independently associated with higher odds of feelings of loneliness (odds ratio [OR]: 2.22; 95% CI: 1.35, 3.65; *p* = .002; OR: 1.70; 95% CI: 1.28, 2.25; *p* < .001). Similarly, the odds of loneliness tended to be higher in older adults who frequently posted on Twitter (OR: 14.3; 95% CI: 4.00, 51.2; *p* < .001). There were no significant associations between SNS usage and loneliness among young adults.

**Table 4:** Logistic regression analysis examining the association between SNS usage^†^ and feelings of loneliness^††^.

	Young adults (n = 2543)		Middle-aged adults (n = 3048)		Older adults (n = 2985)	
Independent variables	OR (95% CI)	*p*-value	OR (95% CI)	*p*-value	OR (95% CI)	*p*-value
***Posting***						
** LINE**	0.99 (0.67–1.46)	.956	1.21 (0.94–1.57)	.137	1.04 (0.78–1.39)	.788
** Facebook**	0.58 (0.32–1.07)	.081	0.87 (0.50–1.50)	.620	0.52 (0.15–1.85)	.317
** Twitter**	1.38 (1.04–1.84)	.026	2.22 (1.35–3.65)	**.002**	14.3 (4.00–51.2)	**< .001**
** Instagram**	1.37 (0.98–1.91)	.067	1.27 (0.70–2.31)	.433	0.63 (0.12–3.44)	.599
**Poor communication**^⁋^	1.32 (1.02–1.70)	.033	1.73 (1.38–2.17)	**< .001**	1.57 (1.20–2.06)	**.001**
***Checking***						
** LINE**	0.95 (0.72–1.26)	.741	1.08 (0.87–1.36)	.481	0.99 (0.73–1.34)	.925
** Facebook**	1.04 (0.78–1.37)	.809	1.12 (0.83–1.51)	.464	1.13 (0.60–2.16)	.702
** Twitter**	1.31 (1.03–1.66)	.028	1.70 (1.28–2.25)	**< .001**	2.06 (1.07–3.96)	.033
** Instagram**	0.94 (0.73–1.22)	.646	0.78 (0.56–1.08)	.130	0.65 (0.25–1.74)	.397
**Poor communication**^⁋^	1.28 (1.00–1.65)	.051	1.69 (1.35–2.12)	**< .001**	1.53 (1.17–2.00)	**.002**

Significant values after applying a Bonferroni correction are in bold.

^**†**^ A value of 1 was given to frequent usage, 0 to infrequent usage.

^††^A value of 1 was given for ‘having a feeling of loneliness’, 0 was given for ‘not having a feeling of loneliness.

^⁋^ Individuals who had contact less than once a week with others through traditional communication, such as face-to-face and non-face-to-face (e.g., telephone, e-mail, letters) communication. A value of 1 was given for poor communication, 0 for non-poor communication.

Each regression model, which was adjusted for covariates (i.e., gender, age, level of education, living arrangement, presence of chronic disease, subjective health, subjective financial status, frequency of going outdoors, frequency of traditional communication with others, and the level of instrumental activity of daily living [only for the group of older adults]), was performed separately for posting and checking on SNS for each age group. All SNS usage was introduced in the same model as the independent variables.

### Poor traditional communication

Low frequency of traditional communication, that is, poor traditional communication, was associated with lower well-being (Table 2), greater distress symptoms (Table 3), and higher odds of feelings of loneliness (Table 4) across all three age groups; however, only the association between poor traditional communication and feelings of loneliness among young adults was not observed.

## Discussion

### Summarizing the key findings

The present study demonstrated generational and SNS-type-dependent associations between SNS use and mental health. There was no common positive association between SNS usage and mental health, independent of the frequency of traditional communication across each generation. However, a negative association between frequent Twitter usage and greater distress symptoms or higher odds of feelings of loneliness was observed. This result might reflect the nature of Twitter, including its higher level of anonymity. Consistent with previous findings [1, 4, 26], the frequency of traditional communication was widely associated with mental health variables across generations, indicating the importance of traditional face-to-face and non-face-to-face (e.g., telephone, e-mail, letters) interactions. However, independent positive associations of better mental health with Instagram use for young adults, with LINE and Facebook use for middle-aged adults, and with LINE use in old adults were confirmed. The findings of the present study suggest that SNS usage may have both benefits and harms, and it is important to strike a balance between communication with and without SNS usage.

### Prevalence of SNS usage

As far as we know, there have been few reports about the prevalence of each SNS usage in Asia and Western countries, which was obtained using a survey similar to the one used in the present study to define frequent usage among various generations using similar criteria. Since a number of studies have adopted online surveys and the definition of frequency of usage is vague, the prevalence is somewhat biased. For example, an earlier US online survey of individuals aged 18 and older using the question, “Which of the following social media platforms do you use in your spare time?,” reported that 34% and 29% of US Internet users accessed Twitter and Instagram, respectively, and Facebook was ranked first with 78% usage rate [31]. An international survey report published in 2018 by the Japanese Ministry of Internal Affairs and Communications [32] reported that the active use of Facebook and Instagram in Japan was much lower than in other countries (the United States, Germany, and the United Kingdom, ranging from 25.9% to 47.7% for Facebook and from 10.2% to 21.0% for Instagram), whereas those of Twitter were comparable (from 8.0% to 16.3%). Further, the prevalence of LINE usage in Japan is reported to be much higher than that in other countries. Considering that each report implies lower usage of each SNS in Japan (except LINE), despite the differences in the methodologies used to demonstrate prevalence, caution should be exercised and the differences in prevalence should be considered when drawing conclusions from our results.

### LINE

The association of message application use with mental health has been examined in various ways, however, the results are inconsistent due to the differences in the types of application and the methods used to define their usage [13]. We found that people who use LINE frequently had better mental health, corroborating a prior finding that active Facebook Messenger use may have a positive impact on mental health [33]. Although there was no significant association in young adults, those of middle-aged (association with distress symptoms) and older adults (association with well-being) were observed (Tables 2 and 3). This implies that even middle-aged and older adults who did not (could not) perform traditional forms of communication with others may maintain their mental health by communicating through a messaging application.

A possible explanation for the conflicting results that only young adults did not show an association between frequent usage of LINE and better mental health may be due to the differences in the prevalence of LINE usage across generations. More than three-fourths of young adults in the present study frequently post on LINE. This result indicates that LINE is an available and familiar SNS tool among young adults, and thus, its usage might be too simple to show an association with their mental health. Considering that poor traditional communication was associated with worse mental health in young adults, they might need traditional communication, including face-to-face conversation, in addition to messenger applications, to maintain social relationships, which can maintain good mental health.

### Facebook

A significant association between frequent posting, but not checking, on Facebook and better well-being was observed only in middle-aged adults (Table 2). This indicates that middle-aged adults need active communication through Facebook, and passive communication is not enough to maintain that in a better way. This result extends prior findings that only active communicators on Facebook pages showed high levels of received social support [34] and maintenance of their mental health, whereas passive Facebook use led to decreased affective well-being [35].

On the contrary, young adults and older adults did not show an association between Facebook use and mental health, indicating that young and older adults use Facebook as a communication tool, but without showing an influence of maintaining their mental health, unlike the middle-aged adults. Previous studies showed both positive and negative effects of Facebook usage, particularly among young adults [7–9, 12]. These conflicting findings have been considered to be a result of an inverted U-shaped curve relationship between the number of Facebook friends and perceived social support, which relates to mental health [36, 37]. If we had examined the number of Facebook friends, we might have obtained interesting results regarding Facebook usage and mental health in young and older adults as well as middle-aged adults.

### Twitter

No positive effects of frequent usage of Twitter were observed among any of the generations in the present study. On the contrary, frequent usage of Twitter was associated with distress symptoms or feelings of loneliness across all three age groups (Tables 3 and 4). It has been suggested that Twitter is easily used in cyberbullying, ridiculing, and undeserved criticism, which are particularly problematic because of its potential for anonymity and the ease with which a number of others could join in the harassment of victims [38]. A previous survey demonstrated that 28% of Twitter users had experienced cyberbullying [39]. Naturally, not all frequent Twitter users engage in or had experienced cyberbullying, but our results might reflect the nature of Twitter, including its higher level of anonymity.

A previous study comparing the Twitter networks of people expressing loneliness or sadness and those expressing feeling loved and happy showed that people expressing negative emotions on Twitter had smaller networks and notably fewer followers [40]. Although some measure of social support through Twitter has been observed [41], evidence from information credibility perception experiments shows a definite tendency among people to view information shared on Twitter as less credible than the other sources [42]. Feelings of loneliness among frequent Twitter users may thus be attributed to small network size and uncredible information/support owing to its anonymity. This speculation is partly supported by the finding of the present study that a lower frequency of traditional communication was independently associated with feelings of loneliness, providing an indication that another style of social support (or social interaction), including face-to-face support, is needed to prevent loneliness.

### Instagram

A recent report by the Royal Society for Public Health in the UK demonstrated that Instagram is the most detrimental SNS for mental health among young adults [11]. However, our study indicated that young adults who frequently check on Instagram showed fine well-being and low distress symptoms. This result is consistent with the previous findings, which suggest that image-based social media use, such as use of Instagram, decreases loneliness and increases happiness and life satisfaction [43]. The ability of Instagram to reduce and mitigate undesirable psychological states and induce positive ones observed in this and previous studies may be due to the ability of images to facilitate social presence [44], resulting in a feeling of connectedness and happiness, which could be similarly transmitted through image-based networks [43]. Although this interpretation seems plausible, it should be noted that only frequent checking was associated with sound mental health in the present study. It remains a challenge for future research to examine the differences posting and checking on image-based social media.

### Limitations

Our results came from a large sample with multiple generations and provide an opportunity to balance the potential benefits and harms of social media use. However, there are limitations to be considered when interpreting the results. First, the cross-sectional study design precludes our ability to assess causality. Second, although frequent usage of SNS in the present study was identified by the number of days of SNS use, for some types of SNS (e.g., Facebook and Instagram), its time usage might have been important to measure the association with mental health. Some previous studies have indicated the danger of SNS addiction, which arises with long usage times per day [45, 46]. If we had included usage time in measurements for some SNS, we might have found a similar impressive result for age-related differences in the association between SNS usage and mental health. Third, we assessed the feelings of loneliness by using a question on the Likert scale, which is limited to measuring the different levels of feelings of loneliness. The difference in culture could also affect the sensitivity of our questionnaire assessing loneliness because of the nature of the Likert scale. Fourth, similar to other mail surveys, comorbidity was assessed by self-report and not clinically assessed. Finally, caution should be exercised when generalizing our results, as the prevalence of each SNS usage may vary across countries and depend on their culture. The issue of whether our findings can be confirmed in other cohorts must be examined in future studies.

## Conclusion

The present study demonstrated generational and SNS-type-dependent associations between SNS use and mental health. These associations indicate both benefits and harms, and it is important to balance communication with and without SNS usage. Based on the findings of the present study, people need to be equipped with the respective tools and knowledge to not only be able to navigate social media platforms in a positive way, but also in such a way as to promote good mental health. On the other hand, our results can also provide a window on the importance of traditional communication, such as face-to-face conversation. Since SNS usage is likely to change over time, the association between usage and health status should be investigated in future studies.

## Supporting information

S1 Table Questionnaires for SNS usage. (DOCX)

S1 Fig Possession rate of communication devices for SNS purposes across seven generations. *Communication device for non-SNS purpose includes individuals who have only flip phones.
(TIF)

S2 Fig Prevalence of frequent SNS users for each SNS application across seven generations. *Frequent usage of each SNS was defined as usage of more than a few times a week.
(TIF)
