# Supplementary material for: Who is mentally healthy? Mental health profiles of Japanese social networking service users with a focus on LINE, Facebook, Twitter, and Instagram
Source: PLoS One. 2021 Mar 3;16(3):e0246090. doi: 10.1371/journal.pone.0246090 (PMC7928453; doi:10.1371/journal.pone.0246090)
Supplement: S1 Table — (DOCX) [file pone.0246090.s001.docx]

**Q. Which of the following communication devices do you have? (please select all that apply)**

| 1　None | 2　Flip phone |
| --- | --- |
| 3　Smartphone | 3 Tablet |
| 4　Computer |  |

**Q. How often do you perform the following SNS activities?**

|  |  | **Every day** | **A few times a week** | **A few times a month** | **Never** |
| --- | --- | --- | --- | --- | --- |
| **(1)** | **Sending messages on LINE** | **1** | **2** | **3** | **4** |
| **(2)** | **Receiving messages on LINE** | **1** | **2** | **3** | **4** |
| **(3)** | **Posting messages on Facebook** | **1** | **2** | **3** | **4** |
| **(4)** | **Checking what others post on Facebook** | **1** | **2** | **3** | **4** |
| **(5)** | **Posting messages on Twitter** | **1** | **2** | **3** | **4** |
| **(6)** | **Checking what others post on Twitter** | **1** | **2** | **3** | **4** |
| **(7)** | **Posting photos on Instagram** | **1** | **2** | **3** | **4** |
| **(8)** | **Checking photos on Instagram** | **1** | **2** | **3** | **4** |
