# Supplementary figures and images for: Who is mentally healthy? Mental health profiles of Japanese social networking service users with a focus on LINE, Facebook, Twitter, and Instagram
Source: PLoS One. 2021 Mar 3;16(3):e0246090. doi: 10.1371/journal.pone.0246090 (PMC7928453; doi:10.1371/journal.pone.0246090)

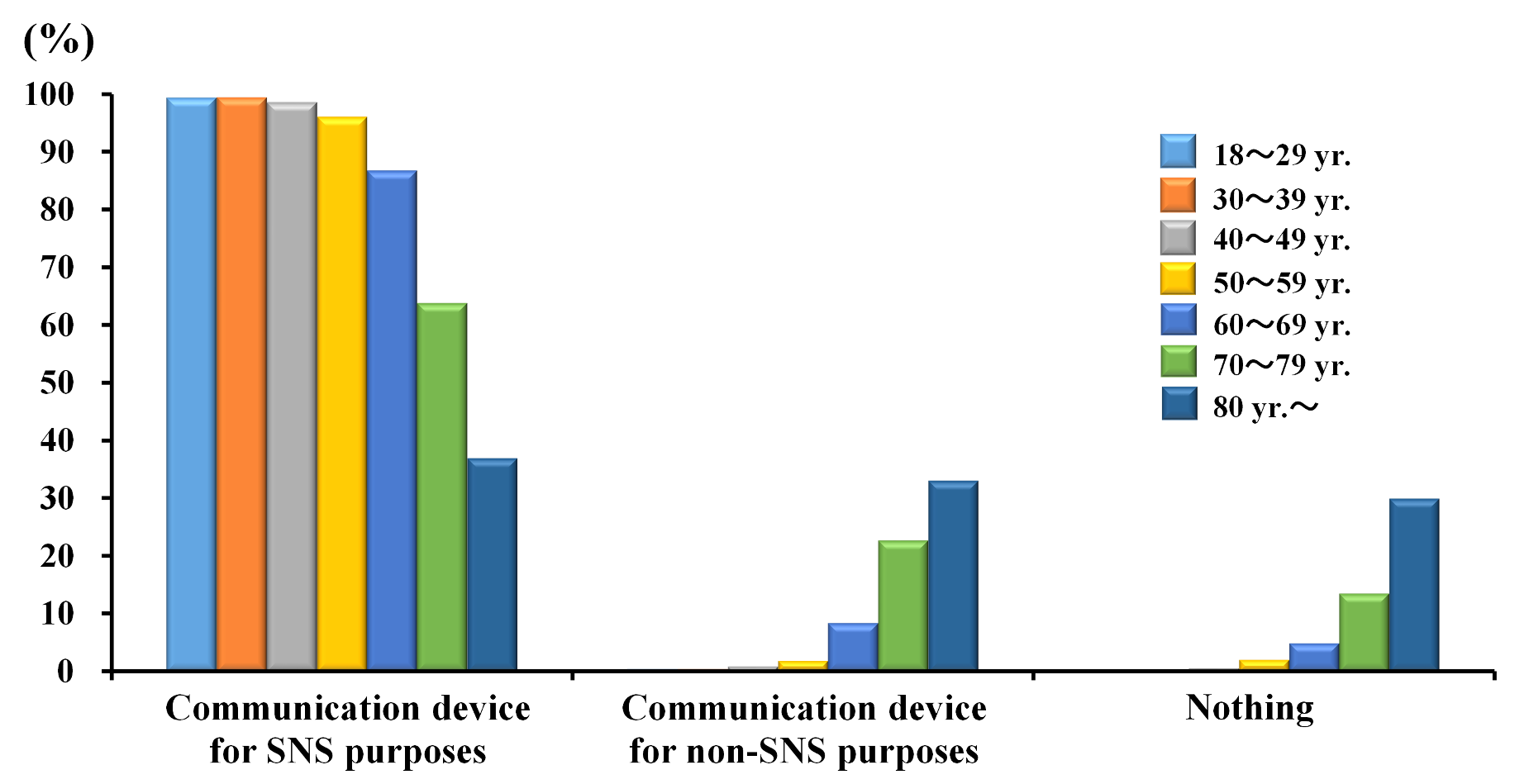

Supplement: S1 Fig — *Communication device for non-SNS purpose includes individuals who have only flip phones. (TIF) [file pone.0246090.s002.tif]

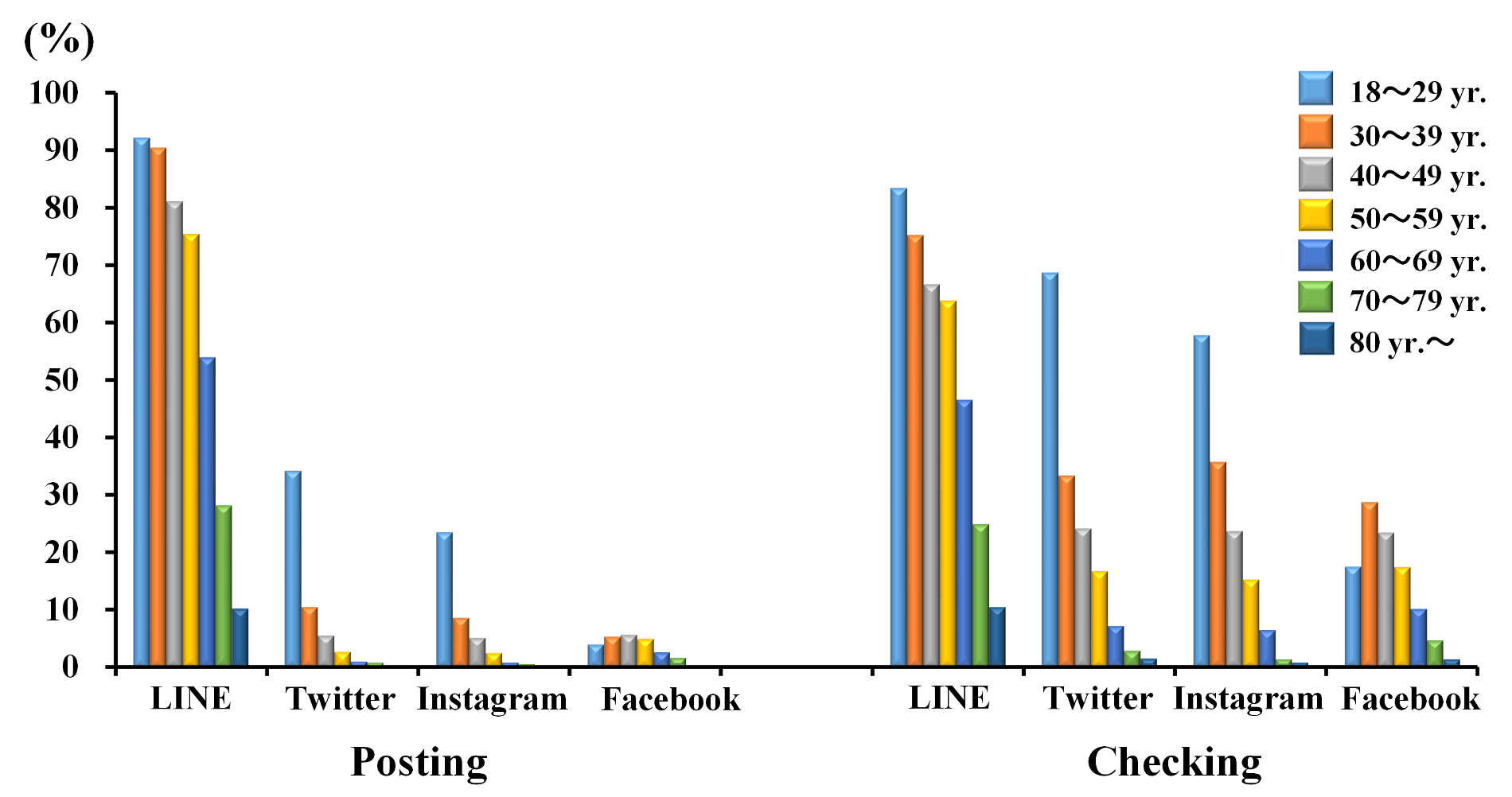

Supplement: S2 Fig — *Frequent usage of each SNS was defined as usage of more than a few times a week. (TIF) [file pone.0246090.s003.tif]
